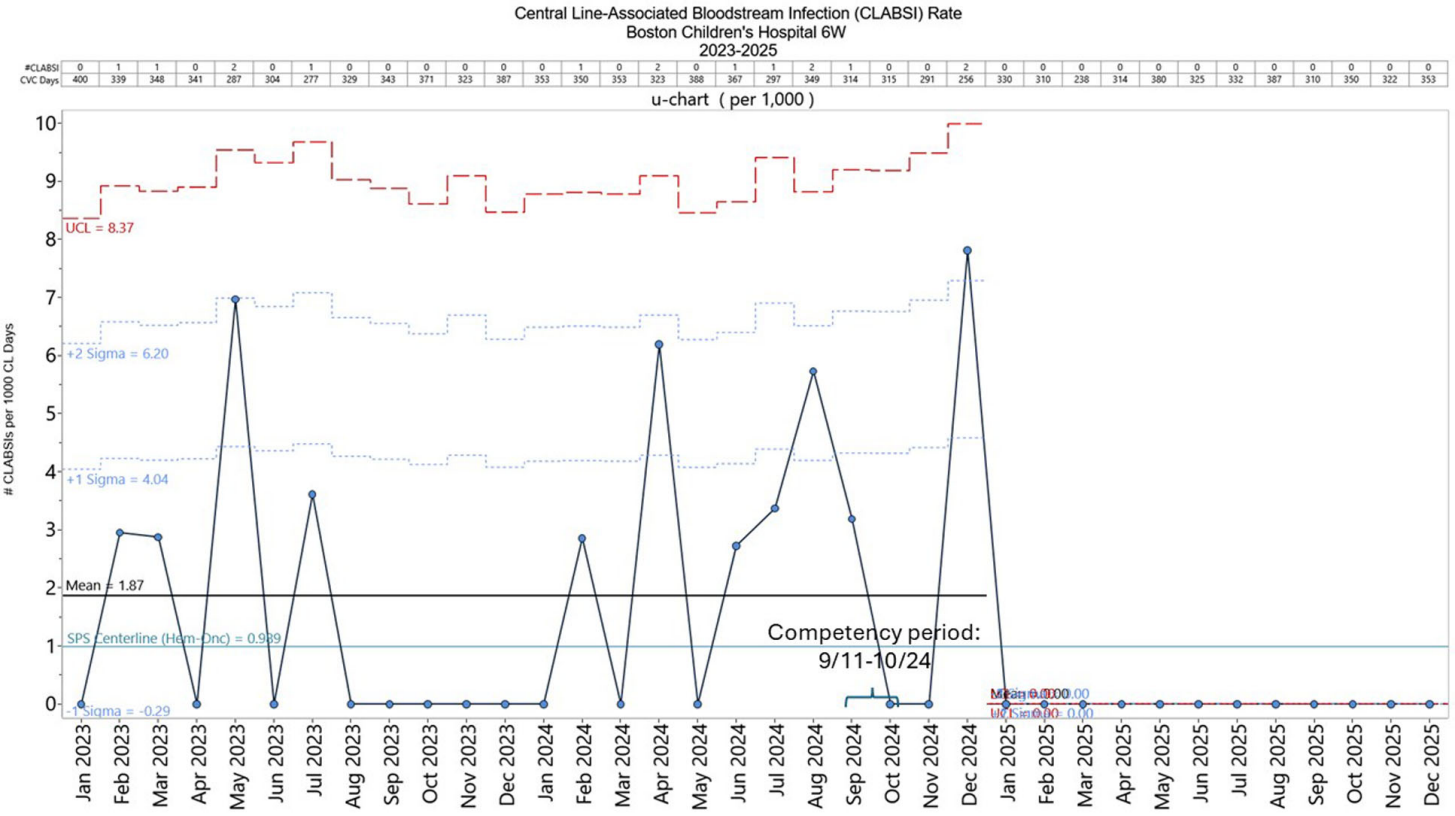# 161 Nontuberculous mycobacteria infections after cardiothoracic surgery with cardiopulmonary bypass among Medicare beneficiaries, 2009–2021

**DOI:** 10.1017/ash.2026.10562

**Published:** 2026-06-23

**Authors:** Christine Hall, Jennifer Ormsby, Paula Conrad, Michelle Connors, Robyn Blacken, Elizabeth Phoung, Cameron Griffin, Jenny Chan Yuen

**Affiliations:** 1 Boston Children’s Hospital; 2 Boston Childrens Hosptial

## Abstract

**Background:** Central line-associated bloodstream infections (CLABSIs) remain a challenge in pediatric care, particularly among medically complex patients requiring prolonged catheterization. Critically ill and immuno-compromised patients maintain high CLABSI rates despite robust efforts to reduce infections. A multifaceted approach to prevention is required, including evidence-based practice standards, competency evaluation, and bundle reliability. Objective: To establish adherence to evidence-based practice standards and evaluate competency. Design/Methods: A structured, multi-unit competency module was developed, emphasizing five core domains of central venous catheter (CVC) care including: sterile dressing changes, timely and aseptic needleless connector changes, standardized use of CVC coverings and securement devices, evidence-based lab-drawing procedures, and skin and environmental hygiene practices; including bathing, room cleanliness and linen management. Staff completed return demonstrations and direct observation audits from March 2024 to March 2025. Units were determined based on NHSN Targeted Assessment Prevention (TAP) report targeting locations with the greatest need. Baseline (January 2023-Febuary 2024) and post-implementation (April 2025 – December 2025) CLABSI rates per 1000 catheter days were measured. Concurrent to the competency, additional interventions included: web-based learning on CVC dressing, chlorhexidine treatment campaign and enhanced visual displays, development and use of high-risk CLABSI reports, and creation of an infection prevention committee in oncology. Results: Three units were identified for the intervention from March 2024 to March 2025, including our medical-surgical intensive care unit (ICU), Hematopoietic Stem Cell Transplant Unit (HSCT), and hematology/oncology. During demonstration, corrections were made on practices, resulting in improved adherence to key CVC maintenance practices, with the most significant improvements seen in dressing and needleless connector change technique and lab-drawing asepsis. From observation, skin and environmental hygiene practices also improved, supporting a need for a comprehensive approach to CLABSI prevention. The overall CLABSI rate for these units decreased from 1.1 pre-intervention to 0.55 post intervention (excluding the intervention period). Most notably, the HSCT unit decreased their CLABSI rate by 100% with zero CLABSIs since intervention resulting in a shift in the mean, and the MSICU saw a 60% reduction. Conclusions: A back-to-basics CLABSI competency and other prevention interventions effectively reinforced essential CVC care practices and improved standardization across inpatient units. Re-establishing core competencies paired with direct observation and environmental hygiene reinforcement, supports sustained reductions in CLABSI risk and strengthens a culture of line safety. Continued monitoring and annual competency refreshers are recommended to maintain practice reliability. Keywords: CLABSI, pediatric oncology, pediatric intensive care, quality improvement, infection prevention

**Figure:**